# Acetate supplementation modulates brain adenosine metabolizing enzymes and adenosine A_2A_ receptor levels in rats subjected to neuroinflammation

**DOI:** 10.1186/1742-2094-11-99

**Published:** 2014-06-04

**Authors:** Mark D Smith, Dhaval P Bhatt, Jonathan D Geiger, Thad A Rosenberger

**Affiliations:** 1 Department of Basic Sciences, University of North Dakota School of Medicine and Health Sciences, 501 North Columbia Road, Grand Forks, North Dakota 58203, USA

**Keywords:** acetate, adenosine, adenosine kinase, adenosine A_2A_ receptor, brain, ecto-5’-nucleotidase, glyceryl triacetate, lipopolysaccharide, neuroinflammation

## Abstract

**Background:**

Acetate supplementation reduces neuroglia activation and pro-inflammatory cytokine expression in rat models of neuroinflammation and Lyme neuroborreliosis. Because single-dose glyceryl triacetate (GTA) treatment increases brain phosphocreatine and reduces brain AMP levels, we postulate that GTA modulates adenosine metabolizing enzymes and receptors, which may be a possible mechanism to reduce neuroinflammation.

**Methods:**

To test this hypothesis, we quantified the ability of GTA to alter brain levels of ecto-5’-nucleotidase (CD73), adenosine kinase (AK), and adenosine A_2A_ receptor using western blot analysis and CD73 activity by measuring the rate of AMP hydrolysis. Neuroinflammation was induced by continuous bacterial lipopolysaccharide (LPS) infusion in the fourth ventricle of the brain for 14 and 28 days. Three treatment strategies were employed, one and two where rats received prophylactic GTA through oral gavage with LPS infusion for 14 or 28 days. In the third treatment regimen, an interventional strategy was used where rats were subjected to 28 days of neuroinflammation, and GTA treatment was started on day 14 following the start of the LPS infusion.

**Results:**

We found that rats subjected to neuroinflammation for 28 days had a 28% reduction in CD73 levels and a 43% increase in AK levels that was reversed with prophylactic acetate supplementation. CD73 activity in these rats was increased by 46% with the 28-day GTA treatment compared to the water-treated rats. Rats subjected to neuroinflammation for 14 days showed a 50% increase in levels of the adenosine A_2A_ receptor, which was prevented with prophylactic acetate supplementation. Interventional GTA therapy, beginning on day 14 following the induction of neuroinflammation, resulted in a 67% increase in CD73 levels and a 155% increase in adenosine A_2A_ receptor levels.

**Conclusion:**

These results support the hypothesis that acetate supplementation can modulate brain CD73, AK and adenosine A_2A_ receptor levels, and possibly influence purinergic signaling.

## Background

In the central nervous system (CNS), adenosine is a potent neuromodulator that regulates sleep, arousal, neuronal excitability, cerebral blood flow and inflammation [1,2]. Under pathological events or increased metabolic demand, adenosine triphosphate (ATP) metabolism and adenosine levels increase with a decrease in the energy charge ratio ((ATP + 0.5*ADP)/(ATP + ADP + AMP)) [3]. Adenosine is described as a ‘retaliatory metabolite’ [4] since it inhibits high energy utilizing processes, reduces the cellular metabolic demand, and restores cellular energy levels. Both adenosine and ATP are ubiquitously present in most organs and tissues and have an important signaling role in inflammation [2,5,6]. Thus, adenosine serves as a link between cellular metabolism and inflammatory signaling.

Adenosine modulates brain activity by binding to G-protein coupled purinergic P1 or adenosine (A_1_, A_2A_, A_2B_, and A_3_) receptors [7]. The CNS effects of adenosine are primarily mediated by inhibitory A_1_ and stimulatory A_2A_ receptors [8]. Activation of A_1_ receptors inhibits neuronal excitability by reducing excitatory neurotransmitter release and offers neuroprotection [1]. While activation of peripheral A_2A_ receptors reduces inflammation, central A_2A_ receptor activation increases glutamate outflow and neuroinflammation [9-11]. Adenosine can be formed both inside as well as outside the cell and distinct cell-specific mechanisms for the elevation of extracellular adenosine levels exist [12]. Under conditions of hypoxia, ischemia, inflammation, nerve stimulation or stress, astrocytes release ATP, which is enzymatically metabolized to adenosine [13]. The rate limiting enzyme in this process is ecto-5’-nucleotidase (CD73) [14]. Adenosine catabolism, on the other hand, is controlled by two enzymes: adenosine deaminase and adenosine kinase (AK). Adenosine deaminase converts adenosine to inosine, which is typically negligible in the central nervous system, although this may change under pathological conditions [2]. Adenosine kinase converts adenosine to AMP following its uptake into the cell via nucleoside transporters [15]. Because adenosine can influence neuronal activity and mediate neuroinflammation, being able to modulate adenosine levels or the receptors involved in purinergic signaling has broad therapeutic potential [16].

A reproducible model of neuroinflammation is generated in rats by the infusion of the bacterial endotoxin lipopolysaccharide (LPS) directly into the fourth ventricle of the brain [17]. In brain, LPS binds to the TLR4/CD14/MD-2 receptor complex [18] that activates neuroglia, reduces cholinergic cell immunoreactivity [19], and increases the expression of the pro-inflammatory cytokine interleukin (IL)-1β [20]. Acetate supplementation, induced with glyceryl triacetate (GTA, 6 g/kg, by oral gavage) reduces LPS-induced neuroglial activation [19,21], IL-1β levels [20], and loss of cholinergic immunoreactivity [19]. Glyceryl triacetate is an effective treatment in rat models of Canavan’s disease [22] and traumatic brain injury [23], possesses growth-arresting properties [24] and is shown to be safe and well tolerated in human trials for Canavan’s disease [25]. Oral administration of GTA increase rat plasma acetate by 100-fold and brain acetyl-CoA levels by 2.2-fold compared to water treated rats [19]. Increase in acetyl-CoA metabolism further increases brain energy stores, reduces AMP levels [26] and increases acetylation of nuclear histones [20,27], which is associated with attenuation of pro-inflammatory cytokine release [20]. In microglia and astrocyte cell cultures, acetate reverses LPS-induced hypoacetylation of histones, attenuates pro-inflammatory cytokines, increases anti-inflammatory cytokine expression, reduces nuclear factor-κB and mitogen-activated protein kinase mediated signaling, and reduces prostaglandin E2 and cyclooxygenase 1 and 2 levels [28-30].

Based on this data, we postulated that acetate supplementation modulates the levels of adenosine metabolizing enzymes and adenosine receptors, which may be a possible mechanism by which GTA exerts its anti-inflammatory and neuroprotective effects. To begin to test this hypothesis, we quantified the ability of GTA to alter brain levels and activity of CD73, and the levels of AK and adenosine A_2A_ receptors. We also examined how protein levels and activity differed using both prophylactic and interventional GTA treatment strategies. Prophylactic acetate supplementation prevented the LPS-induced reduction of brain CD73, increased CD73 activity, and prevented the LPS-induced increase of AK and A_2A_ receptor levels. Interventional GTA treatment increased CD73 similar to the prophylactic treatment, but reduced CD73 activity. Furthermore, in contrast to the prophylactic treatment, interventional GTA increased A_2A_ receptor levels compared to the water-treated controls. These data support the hypothesis that acetate supplementation can modulate adenosine metabolizing enzymes and A_2A_ receptor expression levels in the brain and possibly enhance the effects of endogenous adenosine.

## Methods

### Reagents

Glyceryl triacetate, buffer reagents, β-glycerophosphate, erythro-9-(2-hydroxy-3-nonyl) adenine, α, β-methyleneadenosine 5’-diphosphate, and 2-mercaptoethanol were purchased from Sigma (Sigma, St. Louis, MO, USA). A mouse anti-human CD73 antibody and a horseradish peroxidase (HRP)-conjugated goat anti-mouse IgG antibody were obtained from AbD Serotec (AbD Serotec, Raleigh, NC, USA). A HRP-conjugated goat anti-mouse IgG antibody was purchased from Jackson ImmunoResearch (Jackson ImmunoResearch, Westgrove, PA, USA). A mouse anti-adenosine receptor A_2A_ antibody was obtained from Upstate (Upstate, Billerica, MA, USA). A goat anti-adenosine kinase antibody, mouse anti-α tubulin antibody, HRP-conjugated donkey anti-goat IgG antibody, and HRP-conjugated goat anti-mouse IgM antibody were obtained from Santa Cruz Biotechnology (Santa Cruz Biotechnology, Santa Cruz, CA, USA). Absolute ethanol was from Pharmco (Pharmco, Brookfield, CT, USA).

### Animal studies

This study was conducted in accordance with the NIH Guidelines for the Care and Use of Laboratory Animals (NIH Publication No. 80-23) under an approved University protocol using male Sprague-Dawley rats (Charles River, Wilmington, MA, USA). All rats were allowed to acclimate in our facility for at least seven days prior to inclusion in the study. The surgical implantation of cannulas and the induction of neuroinflammation were performed as described previously [19]. A solution of artificial cerebrospinal fluid (aCSF) or LPS (20.0 ng/μL, E coli, 055:B5, Sigma, St. Louis, MO, USA) dissolved in aCSF (Harvard Apparatus, Holliston, MA, USA) was infused continually at a rate of 0.25 μL/hr via the osmotic mini-pump for a period of 14 or 28 days [31,32]. The rats were divided into three separate experiments: a 14- and 28-day prophylactic treatment study, and a 28-day interventional treatment study. During the 14- and 28-day prophylactic treatment studies, rats were treated daily with water or glyceryl triacetate (GTA) at a dose of 6 g/kg by oral gavage. In the 28-day interventional study, rats did not begin receiving daily doses of either water or GTA until 14 days after the start of the LPS infusion. Interventional treatment was started on day 14 due to neuroglia activation being significantly elevated above controls at this time [17].

### Extraction of rat brain tissue

Rats were anesthetized with isoflurane, euthanized by decapitation, and then the brains were removed and dissected anterior to the middle carotid artery in the coronal plane [32]. The postmortem interval for the sample extraction did not exceed 1.5 min. The dissected brains were placed in a tube containing 3 mL of ice cold extraction buffer (50 mM Tris buffer (pH 7.4) containing 150 mM sodium chloride, 1 mM EGTA, 1 mM sodium orthovanadate, 5 mM zinc chloride, 100 mM sodium fluoride, 1 mM PMSF, one complete, EDTA-free tablet (Roche Applied Science, Indianapolis, IN, USA) per 50 mL, and 0.1% Igepal CA-630). The sample was allowed to sit on ice for 10 min, then homogenized using probe sonication. Homogenized samples were centrifuged at 4°C for 20 min at 4,500 x g and the cytosolic portion was aliquotted into small volumes and stored at -80°C until use.

### Western blot analysis

Samples were prepared by boiling in loading buffer composed of 95% Laemmli sample buffer containing 5% 2-mercaptoethanol. The separation was performed on 50 μg of protein using a 15% Tris-HCl gel with an electrophoresis separation of 100 volts for 135 min. The protein was transferred onto a 0.45 μm nitrocellulose membrane at 100 volts for 90 min in ice. Primary antibodies against CD73 (1:1,000 dilution), A_2A_R (1:1000 dilution), adenosine kinase (1:200 dilution), and α-tubulin (1:1,500 dilution) were prepared in 20 mM Tris buffer, pH 7.4, containing 150 mM NaCl, 0.05% Tween 20 (TTBS), and 5% nonfat dried milk. The nitrocellulose membranes were incubated with primary antibody overnight at 4°C, then incubated with HRP-linked secondary antibody for 90 min at room temperature. Secondary antibodies goat anti-mouse IgG (1:20,000 dilution), goat anti-mouse IgG (1:10,000 dilution), donkey anti-goat IgG (1:10,000 dilution), and goat anti-mouse IgM (1:10,000 dilution) (Santa Cruz Biotechnology, Santa Cruz, CA, USA) were prepared in TTBS buffer. Protein bands were detected using either SuperSignal™ West Pico or West Femto Chemiluminescent Substrates (Pierce, Rockford, IL, USA) and analyzed in a UVP Bioimaging System equipped with VisionWorks™ imaging software (version 4.5, Upland, CA, USA, http://www.UVP.com). Protein concentration was measured using the Bradford assay [33]. Optical densities of the proteins of interest are normalized to the optical density of the loading control α-tubulin and all the western blot data are expressed as percentage over controls.

### Ecto-5’-nucleotidase activity

Sample brain extracts were diluted in ice cold assay buffer (50 mM Tris buffer (pH 7.4 at 37°C) containing 20 mM β-glycerophosphate and 20 μM erythro-9-(2-hydroxy-3-nonyl) adenine) to a protein concentration of 3.33 μg/μL. Each sample (500 μg protein) was assayed in duplicate, with one assay containing 400 μM α, β-methylene adenosine 5’-diphosphate (AMPCP) in the buffer, as a control to inhibit the activity of CD73, and the other without AMPCP. Samples were pre-incubated at 37°C for 10 min, and the assay was started by adding 1 mM AMP and incubating for 30 min at 37°C. The reaction was stopped with 3 M perchloric acid and placed on ice before being centrifuged at 18,100 × g for 5 min at 4°C. The adenosine formed as a result of AMP breakdown by CD73 was then converted into its fluorescent derivative and quantified using HPLC with fluorescence detection as described [34]. Adenosine levels from the control reaction were subtracted from the experimental reaction to calculate CD73 activity in units of nmol adenosine/mg protein/min.

### Statistical analysis

When comparing two groups, an unpaired t-test with a two-tailed *P* value was used to calculate statistical differences using GraphPad InStat statistical software (Version 3.10, Graph Pad Software, Inc., San Diego, CA, USA, http://www.graphpad.com). When comparing more than two groups, a One Way ANOVA with a Tukey’s post-hoc test was performed using the same statistical software. All results are expressed as means ± SD with significance set at *P* ≤0.05.

## Results

To test the hypothesis that acetate supplementation modulates brain adenosine metabolizing enzymes (CD73 and AK) and adenosine A_2A_ receptor levels, we measured the levels of these proteins and the activity of CD73 in three parallel studies. In studies one and two, rats were subject to neuroinflammation for either 14 or 28 days and received prophylactic acetate supplementation throughout the duration of the experiment. A third study was performed in which a group of rats were subjected to 28 days of neuroinflammation, and acetate supplementation was started interventionally on day 14 following the start of the LPS infusion.

### Fourteen day prophylactic acetate supplementation

**Figure 1:**
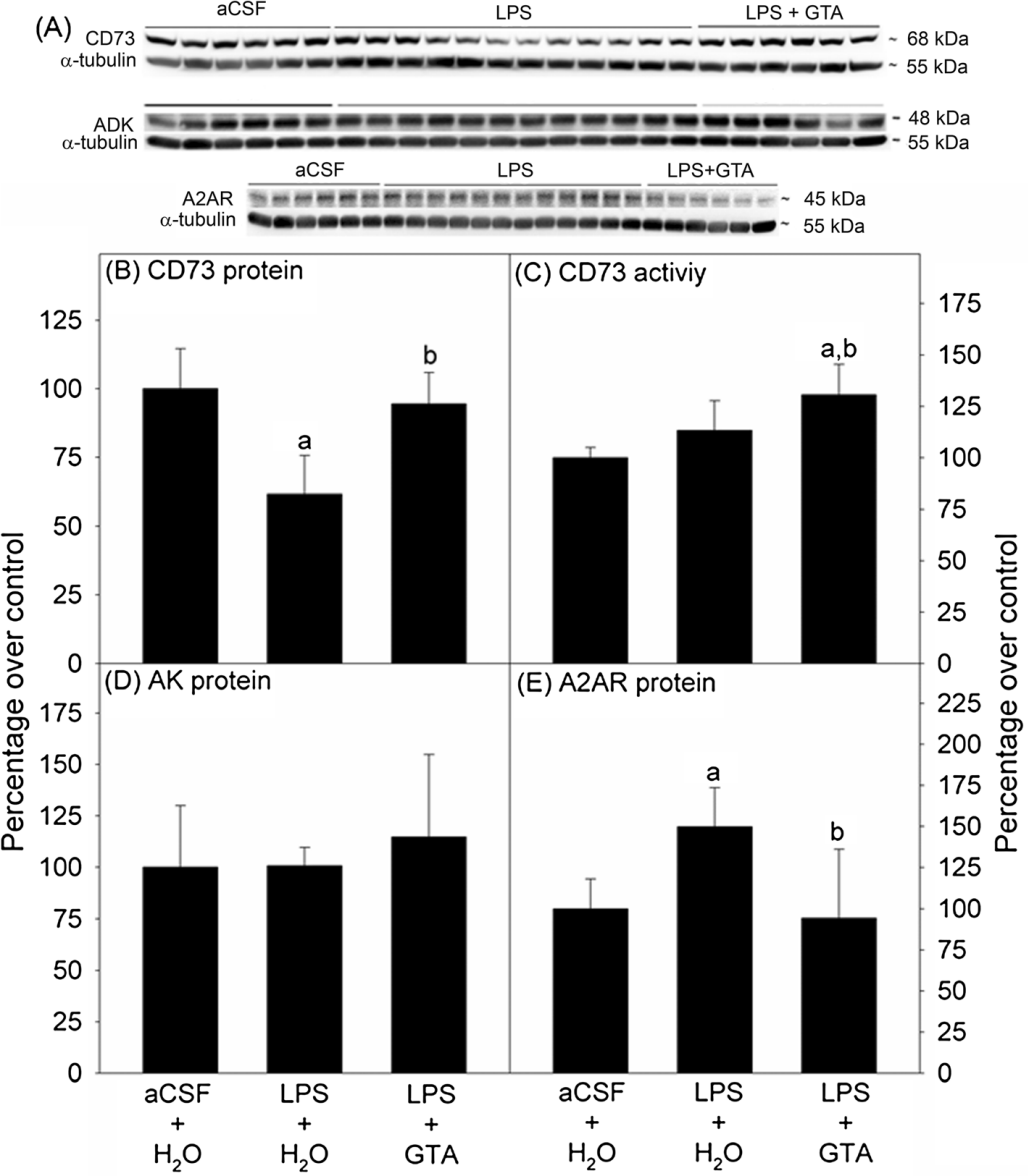
**Fourteen day prophylactic acetate supplementation alters CD73 levels, CD73 activity, and A**_**2A **_**receptor levels.** Panel **A** shows representative images of the western blot analysis for CD73, adenosine kinase (AK), A_2A_ receptor (A2AR), and α-tubulin. Panel **B** and **C** show CD73 levels and CD73 activity, while Panel **D** and **E** show AK levels and A2AR levels, respectively. All values represent the means ± SD, n = 6, 12, 6 for artificial cerebrospinal fluid (aCSF), lipopolysaccharide (LPS) and LPS + glyceryl triacetate (GTA) groups, respectively. Statistical significance set at *P* ≤0.05 and the symbol ‘a’ represents a difference compared to the aCSF group and ‘b’ represents a difference compared to the LPS group.

**Figure 2:**
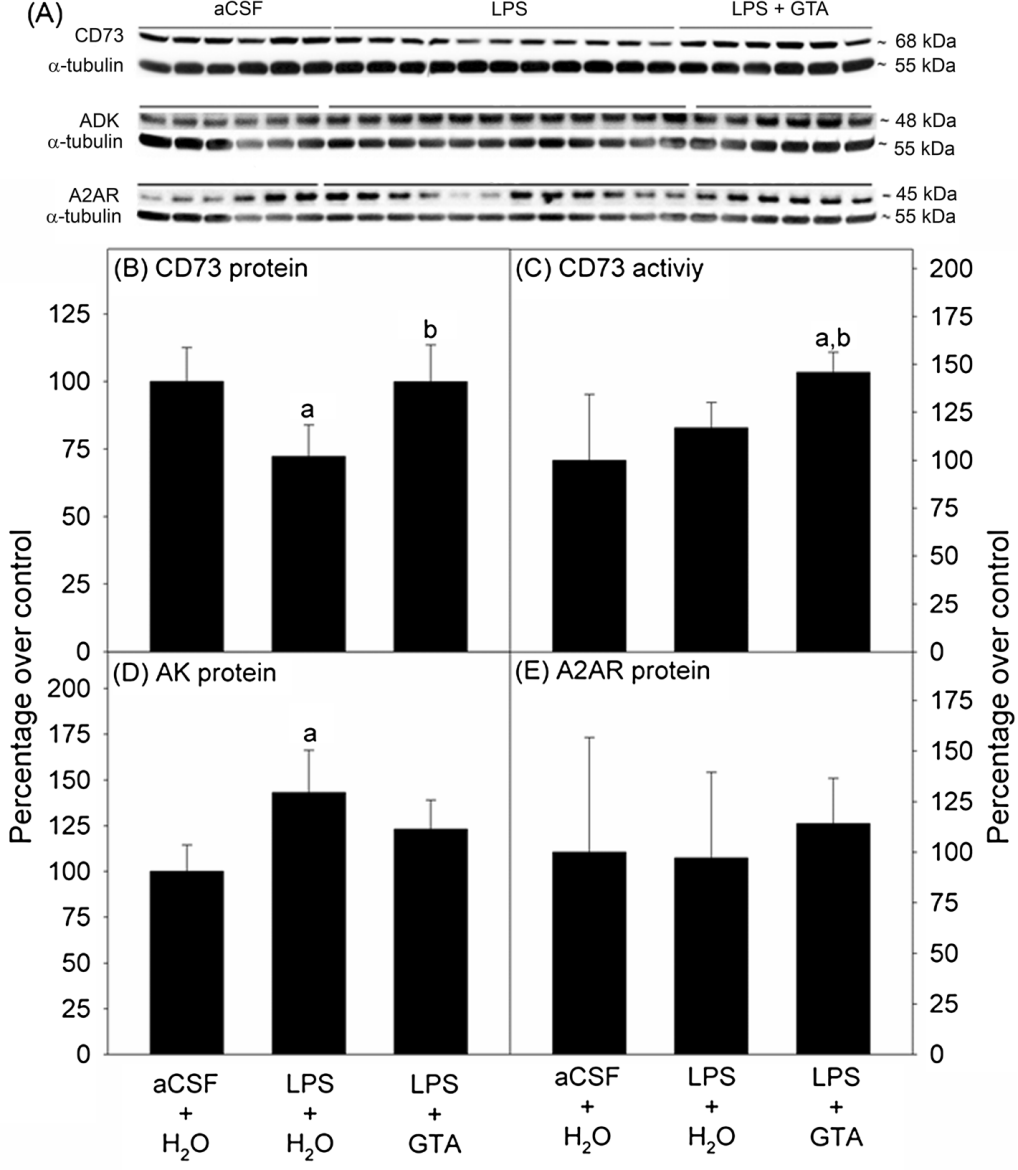
**Twenty-eight day prophylactic acetate supplementation alters CD73 levels, CD73 activity, and adenosine kinase (AK) levels.** Panel **A** shows representative images of the western blot analysis for CD73, AK, A_2A_ receptor (A2AR), and α-tubulin. Panel **B** and **C** show CD73 levels and CD73 activity, while Panel **D** and **E** show AK levels and A2AR levels, respectively. All values represent the means ± SD, n = 6, 12, 6 for artificial cerebrospinal fluid (aCSF), lipopolysaccharide (LPS) and LPS + glyceryl triacetate (GTA) groups, respectively. Statistical significance is set at *P* ≤0.05 and the symbol ‘a’ represents a difference compared to the aCSF group and ‘b’ represents a difference compared to the LPS group.

**Figure 3:**
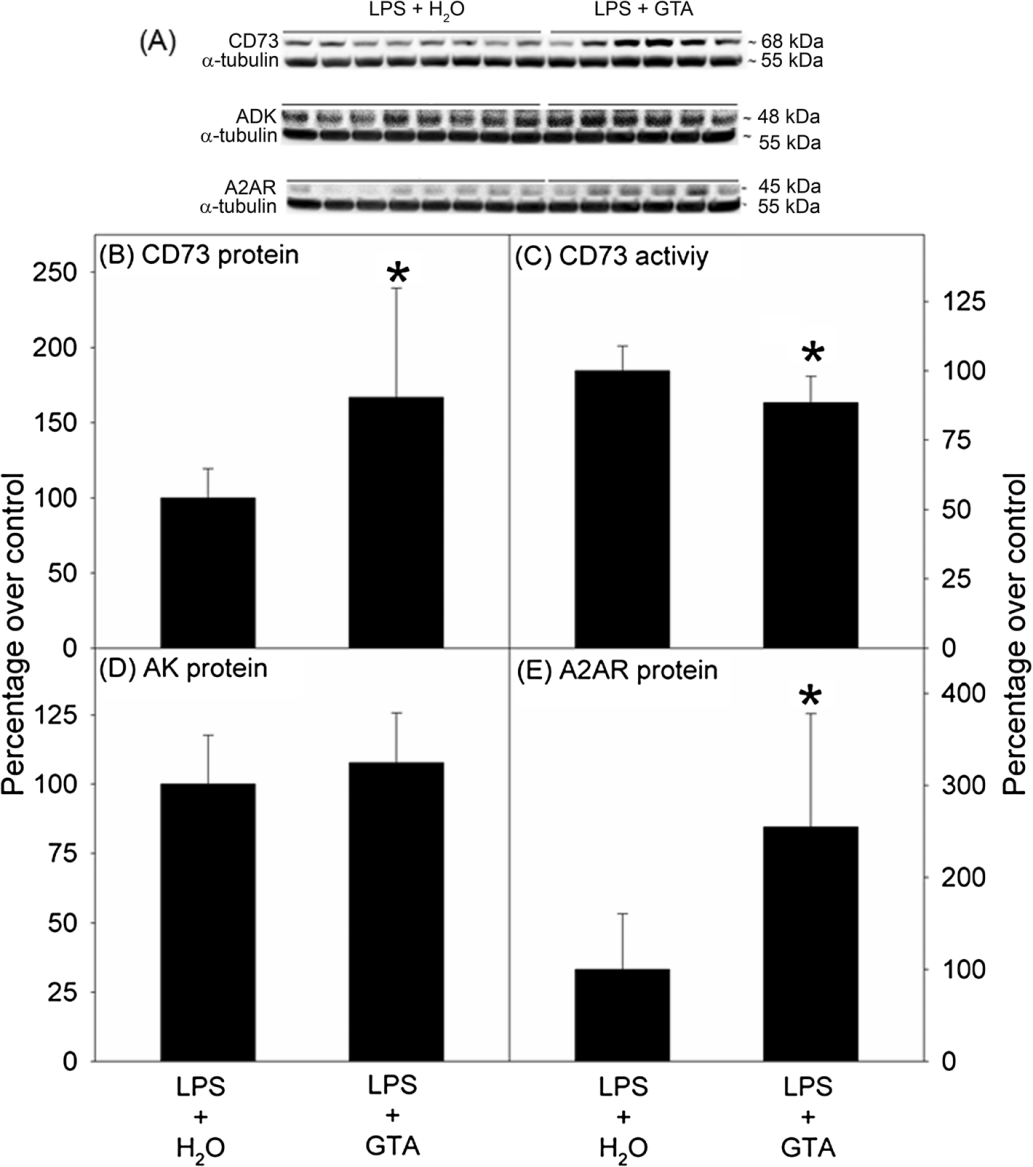
**Interventional acetate supplementation alters CD73 levels, CD73 activity, and A**_**2A **_**receptor levels.** Rats were infused with lipopolysaccharide (LPS) and were divided into two groups; beginning treatment with water or glyceryl triacetate (GTA) beginning on day 14. Panel **A** shows representative images of the western blot analysis for CD73, AK, A_2A_ receptor (A2AR), and α-tubulin. Panel **B** and **C** show CD73 levels and CD73 activity, while Panel **D** and **E** show adenosine kinase (AK) and A2AR levels, respectively. Data represent mean ± SD, n = 8, 6 for LPS + H_2_O and LPS + GTA groups, respectively. The asterisk (*) represents statistical difference compared to LPS + H_2_O group (*P* ≤0.05).

We measured the levels of CD73, AK, and A_2A_ receptor and the activity of CD73 in rats after a 14-day study period. In this study, there were three groups of rats. Group one received sham surgery with aCSF infusion and oral water which served as the control group (n = 6), group two received a LPS infusion dissolved in aCSF with oral water (n = 12), and group three received LPS and were treated with daily oral doses of GTA (6 g/kg body weight) (n = 6). Protein bands for CD73, AK, A_2A_R, and α-tubulin corresponding to molecular weights 68, 48, 45, and 55 kDa respectively, were quantified using western blot analysis (Figures 1A, 2A, and 3A). We found that LPS significantly reduced CD73 levels by 38%, while rats that received LPS plus GTA did not differ from controls (95% ± 11) (Figure 1B). Since CD73 is the rate-limiting enzyme for adenosine formation [14] and changes in its activity are observed in inflammatory conditions [35], we measured CD73 activity in these samples. The activity of CD73 did not significantly differ between control and rats subjected to neuroinflammation. However, rats receiving LPS plus GTA had a significant increase in activity by 31% compared to controls and rats subjected to LPS (Figure 1C). Further, no significant differences in AK levels were observed between the groups (Figure 1D). Based on these data, we measured A_2A_ receptor levels and found that LPS infusion causes a significant increase by 50% compared to controls, while acetate supplementation prevented the LPS-induced increase leaving A_2A_ receptor at control levels (Figure 1E). These results demonstrate that prophylactic acetate supplementation has the capacity to prevent LPS-induced changes in CD73 and A_2A_ receptor levels, and is also able to increase CD73 activity. Although it is not clear whether acetate supplementation achieves an increase in CD73 activity through changes in gene expression or enzyme modification, both may be involved. These data do, however, suggest that acetate supplementation can modulate adenosine metabolizing enzymes and A_2A_ receptor levels.

### Twenty-eight day prophylactic acetate supplementation

A 28-day study was performed to examine the long-term effect of acetate supplementation on brain adenosine metabolizing enzymes (CD73 and AK) and A_2A_ receptor levels. The infusion and treatment groups were identical to those described above except that the GTA treatment and LPS infusion were continued for a total of 28 days. In this study, we found that LPS significantly reduced CD73 levels by 28% of controls, which was not evident in rats that received acetate supplementation (Figure 2B). There was no difference in CD73 activity between controls and rats subjected to neuroinflammation, but LPS-treated rats receiving acetate supplementation showed a significant increase in activity (46%) compared to controls and rats subjected to neuroinflammation (Figure 2C). Twenty eight-day LPS infusion resulted in a significant increase in AK levels (43%) compared to control rats. We found no change in AK levels in rats subjected to neuroinflammation and treated with prophylactic acetate supplementation (Figure 2D). No difference in A_2A_ receptor levels was observed between groups (Figure 2E). These results demonstrate that long-term prophylactic acetate supplementation is able to prevent LPS-induced changes in CD73 and AK levels, and increase CD73 activity using a 28-day prophylactic treatment strategy. Collectively, these studies suggest that neuroinflammation modulates adenosine metabolizing enzymes, which can be prevented with prophylactic acetate supplementation.

### Interventional acetate supplementation

The effect of interventional acetate supplementation (starting at 14 days post-LPS infusion until the 28th day of LPS infusion) on adenosine metabolizing enzymes (CD73 and AK) and A_2A_ receptor levels was examined. Treatment with GTA was begun on day 14 because this is the earliest time when neuroglia activation, based on significant morphological changes in astrocytes and microglia, has been documented [17] in this model. However, the inflammatory signaling starts as early as 6 days following LPS infusion [31]. LPS-treated rats that received an interventional acetate treatment showed a significant increase in CD73 levels (by 67%) when compared to rats that received water (Figure 3B) but demonstrated a significantly lower CD73 activity (by 12%, Figure 3C). There was no significant difference in AK levels between rats receiving water and rats receiving acetate supplementation (Figure 3D). Interventional acetate supplementation resulted in a significant increase in A_2A_ receptor levels (155%) compared to controls (Figure 3E). These results demonstrate that acetate supplementation is able to modulate CD73 and A_2A_ receptor following an interventional treatment strategy. While an increase in CD73 activity was not correlated to an increase in CD73 protein levels as seen with prophylactic treatment, it may be that the mechanism by which acetate exerts its effects takes longer to alter the activity of the enzyme.

## Discussion

Acetate supplementation elevates plasma acetate (approximately 19 mM) [19], brain acetate (approximately 11 mM) [36], and acetyl Co-A (approximately 9 μM) levels [19]. This increase in brain acetyl-CoA metabolism increases brain phosphocreatine levels and reduces AMP levels, suggesting a potential role in altering brain purine metabolism [26]. In order to integrate GTA-induced changes in energy levels [26] with the potential to alter purinergic metabolism, we investigated the effect that acetate supplementation had on purinergic enzymes and receptors in a rat model of neuroinflammation. These studies suggest that acetate supplementation was able to modulate brain ecto-5’-nucleotidase (CD73) and adenosine kinase (AK), as well as the levels of the adenosine A_2A_ receptor, which may describe a mechanism by which acetate exerts its observed anti-inflammatory and potentially neuroprotective effects. The premise that bioenergetic stimulation can influence neuroinflammation has been demonstrated in various animal models of Alzheimer’s disease and Parkinson’s disease, as well as in Parkinson’s disease patients [37]. Acetyl-_L_-carnitine, like acetate, bolsters mitochondrial metabolism in astrocytes and is neuroprotective in a rat model of traumatic brain injury [38]. Similarly, the closest metabolic correlate of acetate supplementation the ketogenic diet, also modulates brain mitochondrial metabolism and is neuroprotective [39,40] through a mechanism involving adenosine [41]. With 14- and 28-day prophylactic acetate supplementation we found that GTA was able to prevent LPS-induced reduction in CD73 levels in addition to increasing CD73 activity (Figures 1 and 2). This suggests that GTA can increase brain adenine nucleotide metabolism and potentially elevate extracellular levels of adenosine. In this regard, interferon-β used for treatment of multiple sclerosis also increases the expression of CD73 [42,43]. Interventional acetate supplementation increased CD73 levels however lower CD73 activity was observed with GTA as compared to the LPS group (Figure 3). Cortical stab wound and focal cerebral ischemia models of brain injury have demonstrated a biphasic alteration in CD73 expression and activity, where an initial decrease is followed by a distinct increase in expression and activity of CD73 during the post-injury period [44-46]. Thus, this suggests that the 14 days of interventional GTA treatment may not be sufficient to elevate CD73 activity. Although during CD73 activity measurements, the non-specific phosphatase activity was inhibited by β-glycerophosphate and the non-CD73 related nucleotidase activity was subtracted from samples by AMCP, involvement of other alkaline phosphatases and intracellular cellular nucleotidases that are not completely inhibited by AMCP cannot be ruled out [47]. Furthermore, different CD73 isoforms in distinct brain regions [47] have been reported which may differentially contribute and interfere with the whole brain CD73 activity analysis in the current study.

Another key enzyme that regulates extracellular levels of adenosine is AK. Although intracellular, the low K_m_ of AK for adenosine (1.5-2.4 μM) [48] combined with the bidirectional and equilibrative nature of adenosine transporters, allows AK to control adenosine uptake. We found that 28 days of LPS-infusion increased AK levels as seen during traumatic brain injury and epilepsy [15,49] (Figure 2). This increase was prevented by the long-term prophylactic treatment, which suggests that GTA in presence of LPS is able to enhance extracellular adenosine availability by preventing the increase in its uptake and conversion to AMP. However, this may lead to a scenario where, elevated adenosine levels become more susceptible to adenosine deaminase mediated degradation to inosine. Since these alterations may be cell-type specific, an in depth *in vitro* analysis of all purinergic enzymes is required to develop a comprehensive view of how acetate alters brain adenosine metabolism.

Extracellular adenosine is an endogenous agonist for four different G protein-coupled (A_1_, A_2A_, A_2B_, and A_3_) receptors, of which the A_1_ and A_2A_ receptors have the greatest relevance in the central nervous system [50,51]. The A_1_ receptor signal cascade generally suppresses neuronal activity, inhibits synaptic transmission, and reduces the activation and response of microglia [52,53]. Adenosine A_2A_ receptors, on the other hand are known to mediate LPS-induced neuroinflammation and neuronal dysfunction [9,10,54]. Antagonists of the A_2A_ receptors have been shown to be anti-inflammatory in a number of CNS disorders [52,54-56], mainly secondary to their inhibitory effects on the glutamate outflow and resulting excitotoxicity [9,10]. In specific regard to our model, it was found that the A_2A_ receptor inhibitor caffeine attenuates LPS-induced neuroinflammation [55]. We found that at 14 days, LPS-induced neuroinflammation caused a significant increase in A_2A_ receptor levels compared to controls (Figure 1), in line with our previous findings that show an increase in IL-1β expression [20], which is known to increase A_2A_ receptor levels [57]. Fourteen-day prophylactic acetate supplementation prevented this LPS-induced increase (Figure 1) in A_2A_ receptors, which is in agreement with results demonstrating that GTA attenuates LPS-induced pro-inflammatory cytokine release [20]. Since blockade of A_2A_ receptor prevents IL-1β induced exacerbation of neuronal toxicity [58], acetate supplementation has a combined effect of reducing IL-1β levels and A_2A_ receptors that can offer robust neuroprotection by attenuating neuroinflammation. However, interventional acetate supplementation unlike the prophylactic treatment increased A_2A_ receptor levels (Figure 3). The reason for this difference is not yet clear, but a bidirectional effect of A_2A_ receptors in neuroinflammation has been described [59]. It is possible that 14 days of interventional acetate supplementation started after significant increases in neuroglia activation have already occurred may not be sufficient to counteract the LPS-induced elevation in A_2A_ receptor levels. However, the ability of the A_2A_ receptors to control neuroinflammation is dependent on local glutamate levels and is critical in determining whether their stimulation results in anti-inflammatory or pro-inflammatory effects [59]. Distinct pathological stages after injury respond to different A_2A_ receptor modulators. For example, during early spinal cord injury A_2A_ receptor agonists offer neuroprotection, while A_2A_ receptor knockout was neuroprotective only during later injury stages [60]. Thus, an alternative reasoning may be that the increase in A_2A_ receptors observed with interventional GTA may be beneficial in enhancing the effects of endogenous adenosine that may contribute towards the anti-inflammatory effect of acetate supplementation. However, to make this determination a thorough investigation of temporal changes in inflammatory markers, A_2A_ receptors, and local glutamate levels with LPS and GTA is required. Further, the protective effects of adenosine A_2A_ receptor agonists and antagonists against spinal cord injury are mediated through their effects in the periphery and the CNS, respectively [11], hence it will be interesting to study how GTA alters peripheral purinergic signaling. A limitation of the interventional study was the lack of the aCSF control group, which would have allowed us to determine whether the increase in A_2A_ receptors was indeed an elevation from control levels. Thus, future studies that examine the effect of interventional GTA strategy for longer treatment duration with adequate controls are necessary. Regardless, this study demonstrates that prophylactic acetate supplementation does prevent the LPS-induced increase in A_2A_ receptor levels, which may help to explain the mechanism by which GTA confers its anti-inflammatory effects.

We previously reported that, acetate supplementation increases histone acetylation [27] and reverses LPS-induced histone H3 at lysine 9 (H3K9) hypoacetylation in a model of neuroinflammation [20]. Histone hyperacetylation alters gene expression and also induces anti-inflammatory effects [61,62]. Therefore, it is reasonable to speculate that acetate induced histone acetylation may be a potential underlying mechanism involved in the modulation of adenosine metabolizing enzymes CD73 and AK as well as the levels of the adenosine A_2A_ receptors, especially at the level of gene transcription. Future studies will determine the link between acetate-induced histone acetylation changes and alteration in the levels of these proteins. Using specific histone acetyltransferase inhibitors to block the effect of acetate on histone acetylation will allow us to determine its role in altering levels of adenosine metabolizing enzymes and receptors. Further, by studying if acetylated H3K9 is associated with the gene promoters of adenosine metabolizing enzyme and receptor will determine if acetate-induced alteration in their expression is controlled by chromatin remodeling.

## Conclusion

Since adenosine is a potent endogenous modulator of neuronal activity and inflammation, development of therapeutic strategies that modulate adenosine metabolism may offer treatment alternatives for neuroinflammation and neurodegenerative disorders. This study attempts to identify the underlying mechanism of action and a treatment regimen for glyceryl triacetate that has been shown to be anti-inflammatory in rat models of neuroinflammation and Lyme neuroborreliosis. In conclusion, both prophylactic and interventional acetate supplementation can modulate adenosine metabolizing enzymes and A_2A_ receptor levels supporting our hypothesis. Future experimentation is needed to determine the specific brain regions, cell types, and mechanisms involved in altering brain adenosine metabolism with acetate supplementation.

## Abbreviations

aCSF: artificial cerebrospinal fluid; ADP: adenosine 5′-diphosphate; AK: adenosine kinase; AMP: adenosine 5′-monophosphate; AMPCP: α,β-methylene adenosine 5′-diphosphate; ANOVA: analysis of variance; ATP: adenosine 5′-triphosphate; A2A receptor: adenosine A_2A_ receptor; A2AR: adenosine A_2A_ receptor; CD73: ecto-5′-nucleotidase; CNS: central nervous system; EDTA: ethylenediaminetetraacetic acid; EGTA: ethylene glycol bis (2-aminoethyl ether)-N, N, N’, N’-tetraacetic acid; GTA: glyceryl triacetate; HPLC: high performance liquid chromatography; IL-1β: interleukin 1β; LPS: lipopolysaccharide; NaCl: sodium chloride; PMSF: phenylmethylsulfonyl fluoride; TTBS: Tris-buffered saline containing Tween 20.

## Competing interests

The authors declare that they do not have competing interests associated with the publication of this manuscript.

## Authors’ contributions

DPB, MDS, JDG, and TAR participated in the research design and wrote or contributed to the writing of the manuscript. The experiments and data analysis were performed by DPB, and MDS. All authors read and approved the final version of the manuscript.
